# Balancing mitochondrial health through inter-tissue neurotransmitter signaling

**DOI:** 10.4103/NRR.NRR-D-25-01191

**Published:** 2026-01-02

**Authors:** Rebecca Cornell, Roger Pocock

**Affiliations:** Development and Stem Cells Program, Monash Biomedicine Discovery Institute and Department of Anatomy and Developmental Biology, Monash University, Melbourne, VIC, Australia

The ongoing health of our bodies is dependent on maintaining a balance between sympathetic and parasympathetic tone. These two branches of the autonomic nervous system work in concert to maintain homeostasis, protect the body against threats, and respond to internal and external stressors. As such, the appropriate and regulated activation of these systems is essential for maintaining health. The concept of balancing excitatory and inhibitory signals is conserved across species to the nematode *Caenorhabditis elegans* (*C. elegans*). Although *C. elegans* lack a distinct autonomic nervous system, they still rely on excitatory and inhibitory neurochemical signaling to mediate behavioral and biochemical processes. Two key players in this balancing act are the excitatory neurotransmitter, acetylcholine, and the inhibitory neurotransmitter, gamma-aminobutyric acid (GABA). In our recent work, we have shown that coordination of these systems is essential for maintaining mitochondrial health across the body (Cornell et al., 2024).

Mitochondria are multifaceted organelles, with functions spanning energy production, pathogen defense, ion buffering, and inter- and intra-cellular signaling. When mitochondria become damaged due to factors such as toxins, misfolded protein aggregates or metabolic challenges, cells must effectively combat the damage to maintain homeostasis. They do this by launching a transcriptional program known as the mitochondrial unfolded protein response (UPR^mt^). The UPR^mt^ boosts chaperone protein production to refold or destroy misfolded proteins, with the aim of restoring cellular proteostasis. The UPR^mt^ is highly conserved and plays a key role in buffering cells against stressors linked to aging, metabolic dysfunction and neurodegenerative diseases.

Traditionally, the UPR^mt^ was considered to be a strictly cell-autonomous process. However, seminal studies using *C. elegans* have revealed key roles for the nervous system in coordinating mitochondrial stress responses in distal tissues, which are not themselves experiencing mitochondrial stress. For example, expression of polyglutamine aggregates in neurons leads to the activation of the UPR^mt^ in intestinal cells (Berendzen et al., 2016). Communication of this signal is dependent on the Wnt signaling ligand, EGL-20, which is both necessary and sufficient to induce the cell non-autonomous UPR^mt^ in intestinal cells (Zhang et al., 2018). Additional neuropeptides that are required for full distal UPR^mt^ induction include insulin-like peptides INS-17 and INS-34, neuropeptide-like proteins NLP-10 and NLP-28, and FMRF-like peptide FLP-15 (Shao et al., 2016). Intriguingly, these studies all showed that serotonin is required for stress response induction in intestinal cells, although serotonin itself is not sufficient to induce the systemic UPR^mt^ (Berendzen et al., 2016; Shao et al., 2016; Zhang et al., 2018). In fact, prior to our study, no chemical neurotransmitter had been found to induce a systemic mitochondrial stress response.

**Figure 1:**
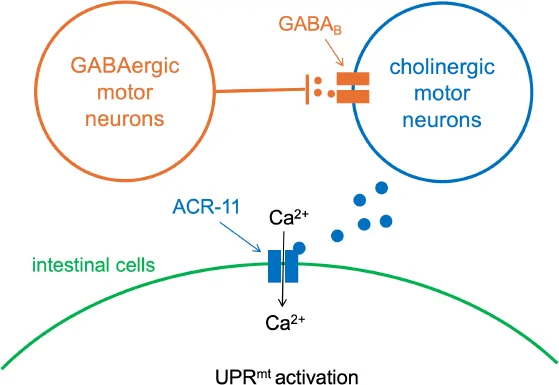
Model of GABA/acetylcholine control of systemic UPR^mt^ activation. GABA released from D-type GABAergic motor neurons binds to the inhibitory GABA_B_ receptor complex on cholinergic motor neurons. When this process is disrupted, it leads to increased acetylcholine release. Acetylcholine activates the intestinally expressed nicotinic acetylcholine receptor, ACR-11, which leads to calcium ion influx and UPR^mt^ activation (Cornell et al., 2024). GABA: Gamma-aminobutyric acid; UPR: unfolded protein response.

**Acetylcholine and GABA balance systemic UPR**^**mt**^ **activation:** A major challenge in neurobiology is understanding how the static nervous system can mount adaptive and systemic responses to physiological stressors. Neuropeptides have been extensively reported to coordinate systemic stress. However, surprisingly, a role for chemical neurotransmitters in coordinating systemic stress responses has remained elusive. In a screen of neurotransmitter mutants, we found that loss of GABA, by mutation of the glutamic acid decarboxylase encoding *unc-25* gene, induces mitochondrial stress responses in intestinal cells. We showed that GABAergic motor neurons control the intestinal UPR^mt^ by regulating acetylcholine signaling through the metabotropic GABA_B_ receptor complex. When the inhibitory GABA signal is lost, cholinergic neurons are overactive, leading to increased acetylcholine release. Supporting a role for acetylcholine in the circuit, increased systemic acetylcholine in the *ace-1*; *ace-2* compound mutant, which is defective in degrading acetylcholine, also induces a mitochondrial stress response in the intestine. Further, we identified an intestinally expressed nicotinic acetylcholine receptor, ACR-11, that is essential for GABA/acetylcholine signaling to regulate the mitochondrial stress response. ACR-11 loss rescues both the intestinal calcium induction and the UPR^mt^ induced by excess systemic acetylcholine, as well as providing resistance to mitochondrial stress in otherwise sensitive mutants. Thus, our research revealed a pathway where GABA/acetylcholine signaling converges on a specific acetylcholine receptor to mediate the UPR^mt^ in distal tissues (**Figure 1**).

**Acetylcholine signaling to the intestine:** Acetylcholine released into synaptic clefts or neuro-muscular junctions is rapidly degraded by acetylcholinesterases (ACE-1 and ACE-2 in *C. elegans*). This process prevents the spillover of acetylcholine and maintains precise spatial and temporal neurotransmission. This makes our finding that the *C. elegans* intestine, a non-innervated tissue, expresses acetylcholine receptors, and that these receptors play a key role in regulating systemic mitochondrial health, particularly surprising. The implication that acetylcholine can persist long enough to act on a distant receptor raises the possibility of non-canonical release routes that bypass synaptic degradation. Long-range or extra-synaptic neurotransmitter signaling has precedent in multiple systems. Volume transmission of acetylcholine occurs within the mammalian central nervous system, particularly by striatal cholinergic neurons (Özçete et al., 2024). This involves extra-synaptic acetylcholine being released and diffusing towards target cells, potentially hundreds of nanometers or even millimeters away, as opposed to synaptic transmission, which occurs across only tens of nanometers (Özçete et al., 2024). Extra-synaptic acetylcholine signaling to non-neuronal tissues has previously been observed in *C. elegans*. During development, acetylcholine secreted from cholinergic neurons regulates the migration of an epithelial cell called the leader cell (Kato et al., 2021). The leader cell follows the worm’s ventral nerve cord, guided by extra-synaptic acetylcholine, which it senses via a muscarinic acetylcholine receptor, GAR-3 (Kato et al., 2021). *In vitro* studies have also shown that non-synaptic acetylcholine can drive cell migration of smooth muscle cells (Li et al., 2004) and mesenchymal stem cells (Tang et al., 2012), via nicotinic and muscarinic receptors, respectively. These examples suggest that, if not degraded, acetylcholine can have sustained effects outside of the synaptic cleft.

An alternative possibility is that acetylcholine may be packaged into extracellular vesicles released by neurons to be trafficked throughout the body. This delivery mechanism is utilized by *C. elegans* to traffic RNAs, signaling proteins and metabolites involved in metabolism and immunity, and is a possible route for extra-synaptic acetylcholine signaling (Russell et al., 2020). Generally, however, the release sites, spatiotemporal scales and biochemical processes underlying non-synaptic neurotransmitter action are poorly characterized. Understanding how acetylcholine reaches and activates intestinal receptors extra-synaptically could open new avenues into brain-gut signaling research.

**Interpreting mitochondrial morphology in the context of stress:** An intriguing challenge that we faced when analyzing our results was the dichotomy of stress resistance outcomes for animals with increased mitochondrial fragmentation. We reported that both mitochondrial stress sensitive (*unc-25*, and *ace-1*; *ace-2*) and resistant (*acr-11*) mutant animals exhibited increased incidence of intestinal mitochondrial fragmentation under basal conditions. Mitochondria are highly dynamic organelles, and a balance between fission and fusion events is essential for maintaining a functional mitochondrial network. Fission is required for mitochondrial biogenesis, as mitochondrial numbers are increased by division, and for clearing damaged mitochondria via mitophagy. Fission can be increased in response to extended periods of stress as part of an adaptive response. For example, long term caloric restriction in a mouse model (40% decrease in all calories over 6 months) increases expression of fission related proteins FIS1 and DRP1, which results in increased mitochondrial biogenesis, as well as cristae number per mitochondria (Khraiwesh et al., 2013). Fusion of mitochondria allows for cross-complementation, as functional and damaged mitochondria merge to enable metabolite, oxidative phosphorylation component, and mitochondrial DNA sharing, thus preserving mitochondrial function across the whole cell (Silva Ramos et al., 2019; Campbell and Zuryn, 2024). Alterations in mitochondrial morphology can correlate with changes in key mitochondrial functions, including adenosine triphosphate production, reactive oxygen species generation, calcium handling, and communication with other cellular structures, such as the endoplasmic reticulum. Elongated mitochondrial networks resulting from increased fusion events are often associated with enhanced oxidative phosphorylation efficiency and resistance to stress. Conversely, smaller, fragmented mitochondria, which arise from increased mitochondrial fission, can exhibit compromised mitochondrial function. These fragmented mitochondria may present with reduced adenosine triphosphate production, impaired mitochondrial quality control, and changes to intracellular calcium storage, with mitochondria that are less efficient in buffering calcium ions, leading to increased cytosolic calcium levels (Campbell and Zuryn, 2024). These perturbations are likely occurring in intestinal cells with unbalanced acetylcholine signaling. Essentially, in *unc-25* and *ace-1*; *ace-2* mutant animals, which we showed to be sensitive to mitochondrial stress, mitochondrial fragmentation likely represents pathological disruption. Conversely, we proposed that *acr-11* mutants were experiencing mitochondrial fragmentation as a product of enhanced mitophagy, wherein they were clearing damaged mitochondria at an increased rate, accompanied by increased biogenesis to replace lost mitochondrial material. This model would confer mitochondrial resilience despite a fragmented appearance. In mammalian and cell-based systems, PGC1 (peroxisome proliferator-activated receptor γ coactivator) is considered the master regulator of mitochondrial biogenesis. Alongside its downstream targets, including the nuclear respiratory factor NRF1, and mitochondrial transcription factor TFAM, the PGC1 pathway is used as a transcriptional readout of mitochondrial biogenesis. In *C. elegans*, no clear orthologues of PGC1 pathway exist, and therefore, confirmation of mitochondrial biogenesis changes is inhibited by technical roadblocks. Instead, conclusions must be inferred by assessing mitochondrial dynamics, density and clearance from the cell. Therefore, the increased rate of autophagy that we observed in *acr-11* knockout animals supports the theory that mitochondrial turnover is upregulated.

Our dichotomous observations of mitochondrial form and function emphasize the limitations of using mitochondrial morphology as a sole indicator of mitochondrial health. Fragmentation can arise from both damaging and protective processes, which static imaging cannot differentiate. Concurrent analyses of mitochondrial function are therefore required alongside morphological imaging to draw conclusions about mitochondrial health.

**Conclusions and perspectives:** In our recent work, we identified a neuro-intestinal circuit that regulates the mitochondrial stress response, mitochondrial dynamics, and organismal survival. Our results identified that the neurotransmitters acetylcholine and GABA play opposing roles in regulating the UPR^mt^, with acetylcholine activating the response, and GABA repressing it via inhibiting acetylcholine release. We identified a previously uncharacterized, intestinally expressed nicotinic receptor, ACR-11, which controls the UPR^mt^. In response to increased acetylcholine, ACR-11 increases intracellular calcium levels in intestinal cells. In turn, this activates the UPR^mt^, alters mitochondrial dynamics and affects organismal survival to mitochondrial stress. By linking GABAergic inhibition and cholinergic activation to systemic metabolism, the nervous system can tune mitochondrial proteostasis according to activity, feeding or environmental challenges. From an evolutionary perspective, this coordination could optimize energy expenditure and resilience in response to changing environmental conditions.

This work brings a new perspective on how the nervous system controls physiology and stress responses systemically, adding to a growing appreciation of brain-gut communication. By balancing excitatory and inhibitory signaling, the nervous system can regulate intracellular processes, which in turn have strong influences on health. The relationship between cholinergic tone dysregulation and systemic mitochondrial function may be particularly relevant to Parkinson’s disease progression, where dopaminergic neuron loss is traditionally associated with striatal cholinergic hyperactivity. Our finding in *C. elegans* suggests that elevated acetylcholine can impair mitochondrial stress resilience. This raises the possibility that cholinergic overactivity may contribute to both the motor dysfunction and impaired mitochondrial function seen in Parkinson’s disease. Future research into how the principles of autonomic balance extend to mitochondrial and metabolic health may be a promising avenue to promote resilience in aging and disease.

*This work was supported by National Health and Medical Research Council grants GNT1105374, GNT1137645 and GNT2000766 (to RP).*
